# Educational outreach visits to improve knee osteoarthritis management in primary care

**DOI:** 10.1186/s12909-019-1504-3

**Published:** 2019-03-01

**Authors:** David Spitaels, Rosella P. M. G. Hermens, Frank P. Luyten, Hilde Vandenneucker, Bert Aertgeerts, Sabine Verschueren, Dieter Van Assche, Patrik Vankrunkelsven

**Affiliations:** 1 Academic Center for General Practice, KU Leuven, Kapucijnenvoer 33, J building, 3000 Leuven, Belgium; 2 Radboud Institute for Health Sciences (RIHS), IQ Healthcare, Radboud University Medical Center, Nijmegen, Netherlands; 3 Division of Rheumatology, University Hospitals Leuven, Leuven, Belgium; 4 Division of Orthopedic Surgery, University Hospitals Leuven, Pellenberg, Belgium; 5 Department of Rehabilitation Sciences, KU Leuven, Heverlee, Belgium

**Keywords:** General practice, Quality indicators, Continuous medical education, Professional training, Physical therapy, Knee osteoarthritis

## Abstract

**Background:**

Knee osteoarthritis is a common problem, but often underdiagnosed and undertreated in primary care as compared to evidence-based guidelines. Educational outreach visits are an effective strategy to improve guideline adherence, but its contribution to knee osteoarthritis management is largely unknown. The aim of this study was to evaluate the overall effectiveness of educational outreach visits on process quality indicators for knee osteoarthritis management, more specifically on the referral for physical therapy.

**Methods:**

An educational intervention study, non-randomized and controlled, was designed for general practitioners (GPs) in Belgium. During four months, 426 GPs were visited by academic detailers and allocated to the intervention group. The control group was selected from GPs not visited by academic detailers during the study period. Six months post-intervention, both groups received a questionnaire with two case-vignettes to measure the effectiveness of the educational outreach. Outcomes were assessed with a Belgian set of quality indicators for knee osteoarthritis management and focused on the number of prescriptions for appropriate physical therapy (i.e. muscle strengthening, aerobic, functional or range of motion exercises) and the adherence to eight additional quality indicators related to knee osteoarthritis management. For the analysis, multivariable logistic regression models were used and Generalized Estimating Equations to handle the correlation between the multiple results per GP.

**Results:**

The intervention group showed a tendency to prescribe more frequently at least one appropriate physical therapy for a case (43.8%), compared to the control group (31.3%, *p* = 0.057). Muscle strengthening exercises were the most frequently prescribed therapy with 37.0% in the intervention versus 26.9% in the control group. The adherence to the other quality indicators showed no significant difference between the intervention and control group and varied between 8.9 and 100% in the intervention group.

**Conclusions:**

This intervention did not alter significantly the adherence to quality indicators and in particular the probability of prescribing physical therapy. To change general practitioners’ prescription behavior, more extensive or combined interventional approaches seem warranted.

**Electronic supplementary material:**

The online version of this article (10.1186/s12909-019-1504-3) contains supplementary material, which is available to authorized users.

## Background

Osteoarthritis is in the top ten list of most frequently managed problems by general practitioners (GPs) [1]. Knee osteoarthritis is the most common form of osteoarthritis and a leading cause of pain and impaired function [2]. The lifetime risk to develop symptomatic knee osteoarthritis is 45%, and nearly 40% of persons above 65 years of age have symptomatic osteoarthritis [3, 4]. With aging of the population and obesity trends, which are both risk factors for osteoarthritis, knee osteoarthritis is expected to become a major burden on healthcare systems [5].

Given the lack of a curative drug treatment for knee osteoarthritis, management guidelines focus on strategies to prevent or delay surgical interventions. The main primary care management principles are the provision of patient information, advice on exercise and weight loss were appropriate [6–9]. This exercise may follow physiotherapy referral, which is effective to reduce pain and functional impairment [10–13]. Quality indicators are important tools in assessing and monitoring the quality of care. For this purpose, a Belgian set of 21 process quality indicators for knee osteoarthritis management, based on international evidence-based guidelines, was developed by a multidisciplinary expert panel [14]. This Belgian set of indicators involves recommendations for diagnosis, lifestyle advice, treatment and follow-up in primary care. According to these quality indicators, patients with symptomatic knee osteoarthritis should be referred to a physical therapist for appropriate exercise therapy. The exercise therapy should include at least muscle strengthening, aerobic and functional exercises, and combined with range of motion exercises in case of range of motion restrictions. In Belgium, the referral rate of patients with knee osteoarthritis for physical therapy is low: an electronic health record review of 576 patients with knee osteoarthritis in primary care showed that only 11% of the patients were referred for physical therapy as part of the non-surgical treatment options, indicating poor guideline adherence [15].

A commitment to lifelong learning is a core component of medical professionalism [16]. The slow rate of guideline implementation in healthcare is a longstanding concern. Literature on evidence regarding different guideline implementation strategies showed that there were no strategies with high certainty of evidence [17]. Educational outreach visits, audit and feedback, decision-support systems are some examples of strategies with moderate certainty of evidence. Educational outreach visits involves independent researchers reviewing data on a certain topic, after which they complete a visit that consists of interactive, one-on-one communication with healthcare professionals in their offices [18, 19]. These visits aim to provide evidence-based, noncommercial information. Educational outreach visits have been employed to improve appropriate prescribing by physicians for antibiotics, benzodiazepines, opioids, NSAIDs, acid-peptic disease management and diuretics for hypertension [20]. These visits have also been employed to target behaviors related to the provision of preventive services or disease management. A Cohrane review on educational outreach visits expected a median adjusted risk difference of 5.6% in compliance with desired practice [19]. In Belgium, an educational outreach project to promote appropriate prescription of pain relief medication, had a significant impact and resulted in a 19% increase in odds for the recommended drug [21]. To date, no research has investigated the effect of educational outreach visits on conservative non-pharmacological management options for knee osteoarthritis in primary care.

The aim of this study was to evaluate the overall effectiveness of an intervention with educational outreach visits on GPs’ adherence to quality indicators and in particular the referral of patients with knee osteoarthritis to physical therapists.

## Methods

### Design and setting

An educational intervention study, non-randomized and controlled, was designed for Belgian general practitioners in collaboration with Farmaka (www.farmaka.be). This government-sponsored organization, scheduled educational outreach visits for 7585 GPs of the 8610 registered GPs across Flanders, Belgium [22]. Between September and December 2014, GPs visited by Farmaka were invited to follow the educational outreach package on knee osteoarthritis management. The effect of the intervention was assessed with two case-vignettes six months post-intervention. The Belgian set of quality indicators was used to develop the intervention and to assess the outcomes [14].

### Study participants

All active GPs, who were already scheduled to receive an educational outreach visit during the study period, were assessed for eligibility to be included in the study. During this visit, GPs were informed about the possibility to receive an additional theme about knee osteoarthritis management. GPs willing to participate were allocated to the intervention group. A control group to measure usual care was selected from the GP membership list of the organization. After computerized randomization, the first 800 GPs from the address list were invited to participate in the control group. We aimed to enroll 350 GPs in the intervention and control group to detect an absolute difference of 15% in referral rate for physical therapy, based on a χ^2^-test with 5% significance level, 1:1 allocation and a power of 80, and assuming a withdrawal rate of 70%.

### Procedure

Academic detailers are specially trained pharmacists, nurses or physicians who are educated in the application of adult learning theory and behavior change, as well as in the details of the clinical problem being addressed [23]. In the preparation of this study, five academic detailers of the Farmaka organization, four pharmacists and one nurse, received an additional two-hour training session focused on the guidance of knee osteoarthritis management and taught by an expert in primary care management (PV) [24]. They received a syllabus based on the Belgian set of quality indicators for knee osteoarthritis management [14]. The importance of prescribing exercise therapy on referral notes for physical therapists was stressed during this training. This information was summarized on a leaflet to be distributed to the GPs in the intervention group.

### Educational intervention

GPs in the intervention group received our intervention during a 20-min, face-to-face educational visit, which was scheduled for another medical theme. Our intervention was scheduled at the end of the visit, and was composed of two-components. First, the academic detailers told the GPs about the evidence-based diagnostic and conservative strategies for knee osteoarthritis management. They particularly focused on the importance of referring symptomatic patients to physical therapists for appropriate exercise therapy. GPs were taught to prescribe a combination of exercise therapy with aerobic, muscle strengthening, functional exercises and range of motion exercises on the referral note for the physical therapist. At the end of the educational visit, the GPs received a printed leaflet, based on the set of quality indicators that summarized the evidence-based diagnostic and conservative management options (Additional file 1) [14].

### Data collection

Through a web-based questionnaire, GPs’ knee osteoarthritis management was assessed in the intervention and control group, six months post-intervention. GPs’ characteristics were collected on: age, gender, practice type (solo/duo/group) and years of experience.

To measure the outcome parameters, two case vignettes were developed: the first case described a patient with recent pain related to knee osteoarthritis, the second case a patient with chronic established knee osteoarthritis (Additional file 2). After the prescription of the cases, four multiple choice questions concerning possible diagnostic examinations, movement therapy, pharmacological treatments and the use of braces or taping, were asked to assess GPs’ management. Four types of appropriate prescriptions for physical therapy, based on the Belgian set of quality indicators, were examined through the case vignettes: muscle strengthening, aerobic exercises, functional exercises and range of motion exercises. Both cases had different functional limitations due to their knee osteoarthritis, which should result in a tailored prescription of appropriate exercise therapy. In the first case vignette only the first three exercise therapies should be prescribed, for the second case vignette also range of motion exercises should be prescribed. The GPs could answer all the questions dichotomously with ‘yes’ or ‘no’ responses. The GPs from the intervention group received the questionnaire six months after the intervention. For the control group, GPs were simultaneously invited with the intervention group between March and July 2015. Participants in both groups were offered the incentive to win two cinema tickets. Non-responders received a reminder by mail three weeks later and a final reminder by post six weeks later.

The two case-vignettes were specifically developed for primary care management, pre-tested, reviewed by an expert panel and met development recommendations for vignettes [25]. The questionnaire was pilot tested among ten GPs for representativeness, completeness and clarity. The case vignettes were adjusted in accordance with their remarks and found to represent an adequate case-mix for patients with knee osteoarthritis in primary care. The answer options in the case vignettes were also discussed by a multidisciplinary expert panel that comprised GPs (DS, PV), an orthopedic specialist (HV), a rheumatologist (FL), physical therapists (SVB and DVA) and an expert in implementation research (RH). Questionnaire invitations were primarily distributed by mails that contained a link to the questionnaire or by post if no e-mail address had been given. The survey software (SurveyMonkey Inc., San Mateo, California, USA) logged the responses in a database that could be used for the purposes of exporting statistical analyses. Two reminder notices were sent at two-weeks intervals.

### Statistical analysis

GPs’ characteristics were calculated with descriptive statistics as mean and standard deviation (Std), numbers with percentages (%) or median and Interquartile range (IQR).

For the primary analysis, a multivariable logistic regression model with group (intervention vs control), and case (case 1, case 2) as fixed factors was used to evaluate the impact of the intervention on the probability that at least one type of appropriate exercise therapy was prescribed for a patient in the two case vignettes (yes/no). In this analysis, group and case served as categorical independent variables. The prescription of at least one type of appropriate exercise was the quality indicator that served as the dependent variable. Generalized Estimating Equations (GEE) were used to handle the correlation between the multiple results per GP (two cases, three to four appropriate therapies). Interactions of intervention with type of therapy and case were verified. In a sensitivity analysis, possible differences in patient characteristics between both groups were evaluated and if necessary added in the multivariable model. Besides the effect of the intervention on the prescription of at least one appropriate exercise therapy on case level, also the prescription of specific types of exercise therapy was calculated by therapy and case.

In a secondary analysis, GPs’ adherence to the eight additional quality indicators was measured at the patient (case) level (Table 4). For this analysis, dichotomous answer options in both case vignettes were allocated to eight additional quality indicators eligible for primary care management (Additional file 3). The group (intervention vs control) was defined as a fixed factor and served as categorical independent variable. The eight remaining quality indicators that were identified acted as dependent variables. A similar approach was used as for the analysis of the prescription of physical therapy, using GEE if the quality indicator was relevant for both cases. A Fisher’s exact test was used in case variability was missing. A significance level was set at *p* < 0.05 for all analyses. All analyses were performed using SAS software, version 9.4 of the SAS System for Windows.

## Results

### Participants

In the intervention group, 17.1% (*n* = 73/426) of the visited GPs returned a completed questionnaire six months post-intervention. Patients in the control group were invited simultaneously with the intervention group to complete the questionnaire. In the control group, 13.1% (*n* = 104/798) of the invited GPs returned a completed questionnaire. Baseline demographic characteristics in the two groups were comparable, with the exception of gender (*p* < 0.019) and practice type (*p* < 0.002): female practitioners were more dominant in the control group (56.3% vs 38.4%) and more GPs worked in group practices in the control group (60.2% vs 37.0%) (Table 1).

**Table 1:** Characteristics of the participating GPs

*Variable*	*Statistic*	*Intervention*	*Control ¥*	*Total*	*P-value*
	N	73	103	176	
Age (yrs)					
	Mean	44.4	41.0	42.4	0.131
	Std	14.35	12.57	13.40	
Gender					
Female	n/N (%)	28/73 (38.36%)	58/103 (56.31%)	86/176 (48.86%)	0.019
Male	n/N (%)	45/73 (61.64%)	45/103 (43.69%)	90/176 (51.14%)	
Practice type					
Solo practice	n/N (%)	30/73 (41.10%)	19/103 (18.45%)	49/176 (27.84%)	0.002
Duo practice	n/N (%)	16/73 (21.92%)	22/103 (21.36%)	38/176 (21.59%)	
Group practice	n/N (%)	27/73 (36.99%)	62/103 (60.19%)	89/176 (50.57%)	
Years of experience					
	Mean	18.2	14.4	16.0	0.107
	Std	14.23	12.25	13.20	
Questionnaire received					
By letter	n/N (%)	11/73 (15.07%)	25/103 (24.27%)	36/176 (20.45%)	0.136
By mail	n/N (%)	62/73 (84.93%)	78/103 (75.73%)	140/176 (79.55%)	

Variables presented with percentages are analysed using a Chi-square test

Variables summarized by means, medians, Std, IQR and Range are analysed using a Mann-Whitney U test

All reported *p*-values are two-sided

¥ Case number 45 in the control group was excluded from this analysis because data on patient characteristics were missing

### Prescription of physical therapy

The probability that for a case at least one type of appropriate exercise therapy was prescribed is higher in the intervention group (*n* = 64/146, 43.8%) than in the control group (*n* = 65/208, 31.3%). Since there was a difference in the distribution of gender and type of practice between both groups, sensitivity analyses were performed extended the model with these two factors. In both models this difference was not significant, neither without (*p* = 0.053, model A), nor with (*p* = 0.057, model B) correction for gender and type of practice (Table 2). Muscle strengthening exercises were the most frequently prescribed appropriate exercise therapy with 37.0% of the GPs in the intervention group prescribing this therapy versus 26.9% the control group. This was followed by functional exercises with 15.1% in the intervention group versus 12.5% in the control group (Table 3). The probability that all appropriate exercise therapies were prescribed for a case was too low to perform statistical analyses, respectively 1/146 (0.7%) in the intervention group and 5/208 (2.4%) in the control group. Note that the effect of intervention did not depend on the specific case (*p* = 0.197 for the interaction between intervention and case).

**Table 2:** Probability that for a case at least one appropriate exercise therapy is prescribed by the GP

		*MODEL A*		*MODEL B*	
		*Odds ratio (95% CI)*	*P-value*	*Odds radio (95% CI)*	*P-value*
Group	Intervention Control	1.72 (0.99; 2.99) #	0.0529	1.77 (0.98; 3.19) #	0.0566 .
Casus^α^	Casus 2 Casus 1	1.38 (1.03; 1.85) #	0.0370	1.39 (1.03; 1.86) #	0.0308 .
Gender	Male Female			0.76 (0.43; 1.36) #	0.3581 .
Type of practice	Duo practice Group practice Solo practice			0.88 (0.39; 1.96) 0.96 (0.47; 1.97) #	0.7449 0.9050 .

α: group and case act as fixed variables

Model A: Results from an additive multivariable logistic regression model with Generalized Estimating Equations to handle the correlation between the multiple results per GP (2 cases). The result is based on 129 events [= total number of positive prescriptions of appropriate therapy in the intervention (*n* = 64) and control (*n* = 65) group] from 354 observations. This model gives the *Odds ratio (95% CI) and p-value* *with no correction* for gender and type of practice

Model B: Same statistical model as for Model A. This model gives the *Odds ratio (95%CI) and p-value* *with additional correction* for gender and type of practice

# reference category

*Note that the effect of intervention did not depend on the specific casus (*p* = 0.1970 for the interaction between intervention and casus)

**Table 3:** Prescription of appropriate exercise therapy by the GP

Type of appropriate exercise therapy	Group	Case	n/N (%)
I. At least one appropriate exercise therapy	Intervention	Case 1 Case 2	31/73 (42.5%) 33/73 (45.2%)
		Total	64/146 (43.8%)
	Control	Case 1 Case 2	27/104 (26.0%) 38/104 (36.5%)
		Total	65/208 (31.1%)
II. Specific type of appropriate exercise therapy			
a. *Muscle-strengthening*	Intervention	Case 1 Case 2	26/73 (35.6%) 28/73 (38.4%)
		Total	54/146 (37.0%)
	Control	Case 1 Case 2	24/104 (23.1%) 32/104 (30.8%)
		Total	56/208 (26.9%)
b. *Aerobic*	Intervention	Case 1 Case 2	3/73 (4.10%) 5/73 (6.80%)
		Total	8/146 (5.50%)
	Control	Case 1 Case 2	4/104 (3.80%) 4/104 (3.80%)
		Total	8/208 (3.80%)
c. *Functional*	Intervention	Case 1 Case 2	10/73 (13.7%) 12/73 (16.4%)
		Total	22/146 (15.1%)
	Control	Case 1 Case 2	11/104 (10.6%) 15/104 (14.4%)
		Total	26/208 (12.5%)
d. *Range of motion*	Intervention	Case 2	9/73 (12.3%)
	Control	Case 2	10/104 (9.60%)

At GP level we saw that after the intervention 39 GPs (53.4, 95% CI: 41.7–65.1) referred for at least one appropriate exercise therapy versus 45 GPs in the control group (43.3, 95% CI: 33.6–53.0). The Fisher’s Exact Test showed that the reference rate was not significant different between both groups (*p* = 0.222).

### Quality indicator adherence

Table 4 shows that for none of the eight additional quality indicators there was a significant difference in adherence between the intervention and control group. In both groups, the advice to complete aerobic and muscle strengthening exercises at home was low with respectively 8.9 and 30.8% in the intervention group, versus 9.6 and 27.4% in the control group. The adherence to the quality indicator, related to the referral of symptomatic patients with knee osteoarthritis for physical therapy, is 47.3% in the intervention group versus 37.9% in the control group. Again, this difference is not significant (*p* = 0.072). Acetaminophen is the most commonly prescribed medication in the intervention and control group (79.5% versus 84.6%). Strong opioids were not prescribed in both groups to alleviate pain.

**Table 4:** Adherence to the additional eight quality indicators for knee OA management measured with the case vignettes

*Quality Indicator*	*Adherence intervention group* *n/N (%)*	*Adherence control group* *n/N (%)*	*Comparison intervention-control group* *p-value ¥*
A. Diagnosis			
1.If a patient is clinically diagnosed with knee OA and suffering from pain resistant to conservative treatment with acetaminophen and/or NSAID, a/ CT scan should not be used.Ω	144/146 (98.6%)	206/208 (99.0%)	0.720 β
b/ MRI should not be used.Ω	135/146 (92.5%)	194/208 (93.3%)	0.680
2. If a patient with knee OA has a recurrent clinically evident effusion, then he/she should be further assessed (with aspiration and analysis of synovial fluid) in order to differentiate from inflammation caused by other arthritis.	73/73 (100%)	104/104 (100%)	/
B. Lifestyle/education/devices			
3. If a patient has knee OA, then a brace should not be prescribed (except in unicompartmental knee OA with axial deviation).Ω	122/127α (96.1%)	204/208 (98.1%)	0.280 β
C. Therapy			
4. If a patient has knee OA, then exercise therapy should be advised, including at least:			
a/ muscle strengthening Ω	45/146 (30.8%)	57/208 (27.4%)	0.986
b/ aerobic exercises Ω	13/146 (8.9%)	20/206 (9.6%)	0.980
5. If a patient has knee OA, then acetaminophen up to 3 g/day should be used as the initial oral analgesic.	58/73 (79.5%)	88/104 (84.6%)	0.353
6. If a patient has knee OA and there is no adequate response on acetaminophen, or there is severe pain and/or inflammation, then oral NSAID should be used.	29/73 (39.7%)	42/104 (40.4%)	0.784
7. If a patient has knee OA, then chondroitin and glucosamine-chondroitin combination products should not be used.Ω	139/146 (95.2%)	192/208 (92.3%)	0.280
8. If a patient has knee OA, then strong opioids (oxymorphone, oxycodone, fentanyl, morphine sulfate) should not be used.Ω	146/146 (100%)	206/206 (100%)	/

Ω The quality indicator is relevant for both cases. The quality indicator adherence is calculated as the sum for both cases

¥ Results from an additive multivariable logistic regression model using Generalized Estimating Equations (GEE) to handle the correlation between the two results per GP if the quality indicator was relevant for both cases

β A regression models using GEE could not be used due to lack of variability. Only the result of a Fishers exact test is reported

α missing values: *n* = 19

## Discussion

### Summary

Our results suggest that educational outreach visits for GPs showed a positive tendency but no significant change in prescribing appropriate physical therapy for patients with knee osteoarthritis. The probability to prescribe for a case at least one appropriate exercise therapy tended to be higher in the intervention group (43.8%), compared to the control group (31.1%). No significant changes were found in the adherence to quality indicators for diagnostic investigations, lifestyle advice and prescription of medication.

Although physical therapy decreases pain and improves function among patients with knee osteoarthritis, it still remains an under-utilized modality [26]. The physical therapy prescription by the GPs in our study is comparable with previously published estimates on patient reported use of physical therapy for knee osteoarthritis (39–52%) [27]. The ‘reactive’ management of knee osteoarthritis in primary care can explain why GPs are not eager on prescribing physical therapy. The management in primary care is currently focused on managing established knee osteoarthritis, mostly defined by chronic knee pain. These patients commonly have massive joint damage. Effective treatment with non-surgical interventions is therefore difficult [28]. The set of quality indicators that we used, advises to prescribe physical therapy with a combination of appropriate exercises, but the focus of the optimal exercise program is still under debate [14]. Juhl et al. stated that the optimal exercise program for knee osteoarthritis should have one aim and focus on improving aerobic capacity, quadriceps muscle strength, or lower extremity performance [29]. Our study clearly shows that GPs do not see an added value of mentioning three to four exercise therapies on the prescription for physical therapists, since in the intervention group only in 0.7% all appropriate exercises were prescribed. Nevertheless, we recommend specifying the type of exercise therapy on referral notes for physical therapists to help ensure that their limited time is not spent on treatment options that do not contribute to high-quality care [30, 31].

The pharmacological management of knee osteoarthritis in primary care is dominated both by acetaminophen and NSAID, as they are both recommended in evidence-based guidelines [6, 7, 32, 33]. Guidelines recommend to start with acetaminophen, because NSAIDs have a serious side effect profile. In our study, acetaminophen was still the most prescribed drug for knee osteoarthritis. Although the review by Machado et al. suggested that acetaminophen has little clinical benefit in OA [34]. Verkleij demonstrated that there was no difference in knee pain and knee function between patients taking diclofenac or acetaminophen in primary care settings [35]. If acetaminophen should stay the “first-line’ treatment for patients with a new episode, this should be further investigated [36].

The major strengths of this study are the assessment of knee osteoarthritis management with a set of quality indicators applicable for primary care. The use of a tailored intervention with case-vignettes can change professional practice, although the effects of tailored interventions are often small or moderate [37]. By developing a short intervention, based on two different strategies with educational outreach and leaflets, we hoped to maximize the educational output taking into account GPs’ limited time. This study also has some limitations. The outreach educational visits were randomly scheduled, but only GPs interested in the intervention participated. Known factors that discourage GPs from requesting outreach educational visits are time spent in the office for continuing medical education, physicians’ perception of wasting working time, and education provided by a non-physician [38]. In spite of several reminders, the study was underpowered with a low response in both study groups, which makes it difficult to draw firm conclusions. The limited time spent on the intervention, the focus on detailed physiotherapeutic treatment options rather than on the importance of early diagnosis, are possible explanations for the low response rate. By using case-vignettes, we avoided measure practice performance by self-reportage as this can overestimate guideline adherence, but these paper-and-pencil vignettes lack the opportunity to follow up patients [39].

## Conclusion

This intervention with educational outreach shows only marginal effects on GPs’ prescription of physical therapy with appropriate exercises for patients with knee osteoarthritis. However, the study was underpowered with a low response in both study groups, which makes it difficult to draw firm conclusions. Educational outreach visits have been frequently employed to improve appropriate prescribing medication, but not often to target behaviors related to the provision of preventive services or disease management. To change the GPs prescription towards more ‘exercise therapy’ a more extensive educational outreach package or multi-faceted interventional approaches seem warranted.

## Additional files

Additional file 1: The flyer provided to the GPs of the intervention group during the educational outreach. (PDF 345 kb)

Additional file 2: The questionnaire for the GPs of the intervention and control group with two case vignettes concerning knee osteoarthritis management. (DOCX 19 kb)

Additional file 3: File to explain the calculation of the quality indicators by matching the quality indicators to the corresponding questions in the case vignettes. (DOCX 16 kb)

## Abbreviations

GEE: Generalized Estimating Eqs.
GP: General practitioner
IQR: Inter quartile range
OA: Osteoarthritis
Std: Standard deviation

## References

[1] Cooke G, Valenti L, Glasziou P, Britt H (2013). Common general practice presentations and publication frequency. Aust Fam Physician.

[2] Johnson VL, Hunter DJ (2014). The epidemiology of osteoarthritis. Best Pract Res Clin Rheumatol.

[3] Murphy L, Schwartz TA, Helmick CG (2008). Lifetime risk of symptomatic knee osteoarthritis. Arthritis Rheum.

[4] Peat G, McCarney R, Croft P (2001). Knee pain and osteoarthritis in older adults: a review of community burden and current use of primary health care. Ann Rheum Dis.

[5] Cross M, Smith E, Hoy D (2014). The global burden of hip and knee osteoarthritis: estimates from the global burden of disease 2010 study. Ann Rheum Dis.

[6] McAlindon TE, Bannuru RR, Sullivan MC (2014). OARSI guidelines for the non-surgical management of knee osteoarthritis. Osteoarthr Cartil.

[7] Fernandes L, Hagen KB, Bijlsma JW (2013). EULAR recommendations for the non-pharmacological core management of hip and knee osteoarthritis. Ann Rheum Dis.

[8] Harding PA, Holland AE, Hinman RS, Delany C (2015). Physical activity perceptions and beliefs following total hip and knee arthroplasty: a qualitative study. Physiother Theory Pract.

[9] National Institute for Health and Care Excellence (NICE): Osteoarthritis: care and management (quality standard 87). http://guidance.nice.org.uk/qs87. Accessed 20 Dec 2018.

[10] Jamtvedt G, Dahm KT, Christie A (2008). Physical therapy interventions for patients with osteoarthritis of the knee: an overview of systematic reviews. Phys Ther.

[11] Brosseau L, Taki J, Desjardins B (2017). The Ottawa panel clinical practice guidelines for the management of knee osteoarthritis. Part two: strengthening exercise programs. Clin Rehabil.

[12] Wellsandt E, Golightly Y (2018). Exercise in the management of knee and hip osteoarthritis. Curr Opin Rheumatol.

[13] Fernandopulle S, Perry M, Manlapaz D, Jayakaran P (2017). Effect of land-based generic physical activity interventions on pain, physical function, and physical performance in hip and knee osteoarthritis: a systematic review and meta-analysis. Am J Phys Med Rehabil.

[14] Grypdonck L, Aertgeerts B, Luyten F (2014). Development of quality indicators for an integrated approach of knee osteoarthritis. J Rheumatol.

[15] Spitaels D, Vankrunkelsven P, Luyten FP, et al. Are the conservative treatment options for patients with knee osteoarthritis fully exploited in primary care? A medical record review in Belgian GP practices. Manuscript under review. 2019.

[16] Brennan TA, Horwitz RI, Duffy FD (2004). The role of physician specialty board certification status in the quality movement. Jama..

[17] Fretheim A, Flottorp S, Oxman A (2015). NIPH Systematic Reviews: Executive Summaries. Effect of Interventions for Implementing Clinical Practice Guidelines.

[18] Soumerai SB, Avorn J (1990). Principles of educational outreach ('academic detailing') to improve clinical decision making. Jama..

[19] O'Brien MA, Rogers S, Jamtvedt G, et al. Educational outreach visits: effects on professional practice and health care outcomes. Cochrane Database Syst Rev. 2007(4):Cd000409. 10.1002/14651858.CD000409.pub2 PMC7032679 17943742

[20] Jin M, Naumann T, Regier L (2012). A brief overview of academic detailing in Canada: Another role for pharmacists. Can Pharm J (Ott).

[21] Bruyndonckx R, Verhoeven V, Anthierens S (2018). The implementation of academic detailing and its effectiveness on appropriate prescribing of pain relief medication: a real-world cluster randomized trial in Belgian general practices. Implement Sci.

[22] Annual statistics relating to health professions in Belgium, 2015. http://www.health.belgium.be/nl/gezondheid. Accessed 21 Jan 2019.

[23] Trotter Davis M, Bateman B, Avorn J (2017). Educational outreach to opioid prescribers: the case for academic detailing. Pain Physician.

[24] Habraken H, Janssens I, Soenen K (2003). Pilot study on the feasibility and acceptability of academic detailing in general practice. Eur J Clin Pharmacol.

[25] Gould D (1996). Using vignettes to collect data for nursing research studies: how valid are the findings?. J Clin Nurs.

[26] DeHaan MN, Guzman J, Bayley MT, Bell MJ (2007). Knee osteoarthritis clinical practice guidelines -- how are we doing?. J Rheumatol.

[27] Abbate LM, Jeffreys AS, Coffman CJ (2018). Demographic and clinical factors associated with non-surgical osteoarthritis treatment use among patients in outpatient clinics. Arthritis Care Res (Hoboken)..

[28] Luyten FP, Bierma-Zeinstra S, Dell'Accio F (2018). Toward classification criteria for early osteoarthritis of the knee. Semin Arthritis Rheum.

[29] Juhl C, Christensen R, Roos EM, Zhang W, Lund H (2014). Impact of exercise type and dose on pain and disability in knee osteoarthritis: a systematic review and meta-regression analysis of randomized controlled trials. Arthritis Rheumatol.

[30] Spitaels D, Hermens R, Van Assche D (2017). Are physiotherapists adhering to quality indicators for the management of knee osteoarthritis? An observational study. Musculoskelet Sci Pract.

[31] Peter WF, Nelissen RG, Vlieland TP (2014). Guideline recommendations for post-acute postoperative physiotherapy in total hip and knee arthroplasty: are they used in daily clinical practice?. Musculoskeletal Care.

[32] Hochberg MC, Altman RD, April KT (2012). American College of Rheumatology 2012 recommendations for the use of nonpharmacologic and pharmacologic therapies in osteoarthritis of the hand, hip, and knee. Arthritis Care Res (Hoboken).

[33] Kingsbury SR, Gross HJ, Isherwood G, Conaghan PG (2014). Osteoarthritis in Europe: impact on health status, work productivity and use of pharmacotherapies in five European countries. Rheumatology (Oxford).

[34] Machado GC, Maher CG, Ferreira PH (2015). Efficacy and safety of paracetamol for spinal pain and osteoarthritis: systematic review and meta-analysis of randomised placebo controlled trials. BMJ..

[35] Verkleij SP, Luijsterburg PA, Willemsen SP (2015). Effectiveness of diclofenac versus paracetamol in knee osteoarthritis: a randomised controlled trial in primary care. Br J Gen Pract.

[36] Conaghan PG (2016). NSAIDs or paracetamol for short-term treatment of mild to moderate knee pain in early osteoarthritis: are they equivalent?. Evid Based Med.

[37] Baker R, Camosso-Stefinovic J, Gillies C, et al. Tailored interventions to address determinants of practice. Cochrane Database Syst Rev. 2015(4):Cd005470. 10.1002/14651858.CD005470.pub3 PMC7271646 25923419

[38] Allen M, Ferrier S, O'Connor N, Fleming I (2007). Family physicians' perceptions of academic detailing: a quantitative and qualitative study. BMC Med Educ.

[39] Adams AS, Soumerai SB, Lomas J, Ross-Degnan D (1999). Evidence of self-report bias in assessing adherence to guidelines. Int J Qual Health Care.

