# Supplementary material for: Educational outreach visits to improve knee osteoarthritis management in primary care
Source: BMC Med Educ. 2019 Mar 1;19:66. doi: 10.1186/s12909-019-1504-3 (PMC6397491; doi:10.1186/s12909-019-1504-3)
Supplement: Supplementary file 1 — The flyer provided to the GPs of the intervention group during the educational outreach. (PDF 345 kb) [file 12909_2019_1504_MOESM1_ESM.pdf]

Knee osteoarthritis

KU Leuven – Academic Centre for General Practice
Kapucijnenvoer 33 building J, 3000 Leuven, Belgium

Diagnosis

Symptoms!

- > 40 years + usage-related knee pain+ morning stiffness < 30 min + functional limitation

and

- 1 or more of the following typical clinical findings:
  - crepitus
  - restricted movement
  - bony enlargement

Additional investigations

(X-ray, blood analyses, joint aspiration)

- Patients with suspected knee OA, but who are not eligible for a clinical diagnosis
- Patients with a clinical diagnosis and recurrent swelling of the knee
- Patient with a contra-indication for conservative treatment with acetaminophen ± NSAID or persistent pain → X-ray of the symptomatic knee

X-ray of the symptomatic knee

- weight-bearing
- semi-flexed PA view
- Lateral and skyline view

There is **NO** indication for CT or MRI

Core treatment recommendations

For all patients with knee OA!

1. Education and lifestyle advice

- Information about the treatment objectives and the importance of changes in lifestyle, exercise, weight reduction, and other measures to unload the damaged joints.
- Encourage to lose weight, if there is overweight

2. Exercise therapy

- A combination of muscle strengthening, aerobic and functional exercises
- combined with range of motion exercises in case of range of motion restrictions

→ Refer to **a physical therapist** for instruction in appropriate exercises, for motivation, and to evaluate performance.

3. Pharmacological

- Step 1: acetaminophen up to 3 g/day ± topical NSAID
- Step 2 (no adequate response on acetaminophen or non-pharmacological treatment options): NSAIDs
  - should be used intermittently (max 3 weeks sustained use)
  - at the lowest effective dose

Additional recommendations

Insufficient effect or contra-indications for core treatment options

In function of the X-ray

- Bicompartimental or patellofemoral osteoarthritis
  - Intraarticular injection with corticosteroids (optional)
  - intraarticular injection with hyaluronic acid (optional)
  - Referral to specialist
- unicompartmental knee OA or additional pathology.
  - Referral to specialist (brace, arthroplasty, osteotomy)
