# Supplementary material for: Educational outreach visits to improve knee osteoarthritis management in primary care
Source: BMC Med Educ. 2019 Mar 1;19:66. doi: 10.1186/s12909-019-1504-3 (PMC6397491; doi:10.1186/s12909-019-1504-3)
Supplement: Supplementary file 2 — The questionnaire for the GPs of the intervention and control group with two case vignettes concerning knee osteoarthritis management. (DOCX 19 kb) [file 12909_2019_1504_MOESM2_ESM.docx]

Additional file 2. The questionnaire with two case vignettes about knee osteoarthritis.

# Case 1

A 60-year-old man presents on your consultation with unilateral pain in the knee for several months. Since one week, he experiences activity-related knee pain, especially during walking. This causes inconvenience to do his groceries. The patient has no pain in the affected joint during the night. He has morning stiffness in the painful knee that lasts no longer than 10 minutes. He had no recent (knee) trauma and there is no pain in other joints. He is obese with a Body Mass Index of 30.

In his medical history, he experienced a transient ischemic attack six years ago. He takes Acetylsalicylic Acid 80mg on a daily base.

Clinical examination of the knee reveals crepitus on active motion and pain when pressing on the kneecap. There is a no abnormal clicking or popping when the knee structures are moved. There is no swelling of the joint and full range of motion.

You find the patients’ complaints and physical examination suggestive for knee osteoarthritis.

# Case 2

A 58-year-old woman presents on your consultation with recurrent knee pain. Six months ago, an orthopedic specialist diagnosed her with knee osteoarthritis after an X-ray was performed. The X-ray showed a grade 2 Kellgren Lawrence knee osteoarthritis in the medial and lateral compartment of the affected knee. At that time the specialist prescribed acetaminophen (maximum 3 tablets per day) and local NSAIDs for 10 days. The patient consults with recurrent pain. There is morning stiffness that lasts no longer than 10 minutes. She had no recent trauma.

In the medical history, we also note high blood pressure and normal Body Mass Index of 24 after dietary advice. She takes Bisoprolol 10mg per day.

Clinical examination of the knee reveals crepitus on active motion, slight range of motion restriction, and pain when pressing on the kneecap. There is a no abnormal clicking or popping when the knee structures are moved and no swelling of the joint.

## Question 1: Would you propose further examinations at this stage?

Multiple answers are possible.

1. No examinations
2. X-ray of the knee
3. CAT-scan of the knee
4. MRI of the knee
5. Blood tests
6. Joint aspiration
7. Referral to orthopedic specialist
8. Other: please specify__________

## Question 2: Would you advise movement therapy?

Multiple answers are possible.

1. No movement therapy
2. In the home situation: general movement advice
3. In the home situation: muscle strengthening exercises
4. In the home situation: aerobic training exercises
5. Physical therapy prescription: non-specific referral note
6. Physical therapy prescription: massage
7. Physical therapy prescription: thermotherapy (cold or hot packs)
8. Physical therapy prescription: non-specific exercise therapy
9. Physical therapy prescription: muscle strengthening exercises
10. Physical therapy prescription: aerobic training exercises
11. Physical therapy prescription: functional exercises
12. Physical therapy prescription: range of motion exercises
13. Physical therapy prescription: transcutaneous electrical nerve stimulation (TENS)
14. Physical therapy prescription: electromyostimulation quadriceps
15. Physical therapy prescription: ultrasound
16. Other: please specify_______

## Question 3: Would you propose pharmacological treatment modalities?

Multiple answers are possible.

1. No pharmacological treatment
2. Acetaminophen
3. Topical NSAIDs
4. Oral NSAIDs
5. Intra-articular corticosteroids
6. Intra-articular hyaluronic acid
7. Glucosamine
8. Chondroitin
9. Weak opioids ( codeine, tramadol or combined with acetaminophen)
10. Strong opioids (oxymorphone, oxycodone, fentanyl, morphine sulfate)
11. Other: please specify__________

## Question 4: Do you have additional treatment options?

1. Biomechanical intervention with knee brace
2. Biomechanical intervention with tape
3. Other: please specify_________

# Background variables

## Year of birth?

JJJJ:____________

## Gender?

1. Male
2. Female

## Work setting?

1. Solo practice
2. Duo practice
3. Group practice

## Years of experience as a GP?

__________

## How did you receive this questionnaire?

1. By letter
2. By mail
