# Supplementary material for: Educational outreach visits to improve knee osteoarthritis management in primary care
Source: BMC Med Educ. 2019 Mar 1;19:66. doi: 10.1186/s12909-019-1504-3 (PMC6397491; doi:10.1186/s12909-019-1504-3)
Supplement: Supplementary file 3 — File to explain the calculation of the quality indicators by matching the quality indicators to the corresponding questions in the case vignettes. (DOCX 16 kb) [file 12909_2019_1504_MOESM3_ESM.docx]

Additional file 3. Additional quality indicators and their corresponding questions in the case vignettes.

| Quality Indicator | Corresponding question |
| --- | --- |
| 1. Diagnosis | |
| 1.If a patient is clinically diagnosed with knee OA and suffering from pain resistant to conservative treatment with acetaminophen and/or NSAID,  a/ CT scan should not be used. | Case1:1c and case 2:1c |
| b/ MRI should not be used. | Case1:1d and case 2:1d |
| 2. If a patient with knee OA has a recurrent clinically evident effusion, then he/she should be further assessed (with aspiration and analysis of synovial fluid) in order to differentiate from inflammation caused by other arthritis. | Case1:1f |
| 1. Lifestyle/education/devices | |
| 3. If a patient has knee OA, then a brace should not be prescribed (except in unicompartmental knee OA with axial deviation). | Case1:4a and case 2:4a |
| 1. Therapy | |
| 4. If a patient has knee OA, then exercise therapy should be prescribed, including at least: | |
| a/ muscle strengthening | Case1+:2c and case 2:2c |
| b/ aerobic exercises | Case1:2d and case 2:2d |
| 5. If a patient has knee OA, then acetaminophen up to 3 g/day should be used as the initial oral analgesic. | Case1:3b |
| 6. If a patient has knee OA and there is no adequate response on acetaminophen, or there is severe pain and/or inflammation, then oral NSAID should be used. | Case2:3d |
| 7. If a patient has knee OA, then chondroitin and glucosamine-chondroitin combination products should not be used. | Case1:3h and Case 2:3h |
| 8. If a patient has knee OA, then strong opioids (oxymorphone, oxycodone, fentanyl, morphine sulfate) should not be used. | Case1:3j and case 2:3j |
